# Weak spin-flip scattering in Pd_89_Ni_11_ interlayer of NbN-based ferromagnetic Josephson junctions

**DOI:** 10.1038/s41598-022-10967-6

**Published:** 2022-04-27

**Authors:** Duong Pham, Riku Sugimoto, Kenjiro Oba, Yuto Takeshita, Feng Li, Masamitsu Tanaka, Taro Yamashita, Akira Fujimaki

**Affiliations:** Department of Electronics, Graduate School of Engineering, Nagoya University, Furo-cho, Chikusa-ku, Nagoya, 464-8603 Japan

**Keywords:** Superconducting devices, Superconducting properties and materials, Electrical and electronic engineering, Qubits

## Abstract

We studied niobium nitride (NbN)-based π-junctions with a diluted ferromagnetic Pd_89_Ni_11_ interlayer (NbN/PdNi/NbN junctions). In the NbN/PdNi/NbN junctions with various PdNi thicknesses, we observed a non-monotonic dependence of the critical currents on PdNi thickness, indicating the effects of the exchange interaction on the superconducting order parameter. From theoretical fitting of the experimental data, we found that the NbN/PdNi/NbN junctions showed a significantly smaller degree of spin-flip scattering in the PdNi interlayer than in the CuNi interlayer of NbN/CuNi/NbN junctions reported previously. The weak spin-flip scattering leads to a longer decay length of the Josephson critical current, so the critical currents were observed over a wide range of PdNi thicknesses (10–40 nm). We also fabricated superconducting quantum interference devices (SQUIDs) including the NbN/PdNi/NbN junction, using a PdNi thickness in which the π-state was expected. A half-flux-quantum shift, as evidence of the π-state, was observed in the magnetic field-dependent critical currents of the SQUIDs. This result represents an important step towards the practical application of NbN-based π-Josephson junctions.

## Introduction

In a Josephson junction, two superconductors are coupled by insulating or normal metal interlayers (SIS or SNS junctions), and the superconducting order parameter shows an exponential decay through the interlayers^1^. On the other hand, when the interlayer consists of ferromagnetic materials (SFS junctions), the superconducting order parameter shows spatial oscillation in the ferromagnetic layer due to the interplay between superconductivity and ferromagnetism^2^. Thus, in contrast to conventional junctions, SFS junctions can be in a so-called π-state over a certain range of ferromagnetic layer thicknesses, in which the phase difference between the two superconducting layers becomes π in the ground state. The π-state in an SFS junction (π-junctions) is an interesting topic, not only due to its intriguing physics but also its potential applications.

In the field of quantum computing, superconducting quantum bits (qubits) are proving to be one of the best candidates for building a practical quantum computer^3^. Flux qubits with long coherence times and high anharmonicity have been reported, to facilitate the scaling-up of quantum processors^4^. Conventional flux qubits always require a half-flux-quantum bias to operate at the optimum point for achieving the longest coherence time. However, the necessity of having a half-flux-quantum bias reduces scalability^5^. One way to solve this issue is to insert a *π*-junction into a superconducting loop of the flux-qubit. A coherent two-level state can be created with a zero external magnetic field, so that the flux qubit can be operated optimally without half-flux-quantum bias^6–8^. Recent research into superconducting single-flux-quantum logic circuits has demonstrated the possibility of improving operating margins and energy consumption by introducing π-junctions into these circuits^9–11^. However, despite extensive study of π-junctions for superconducting quantum and classical circuits^2,12–14^, there have been few reports on device implementation of the π-junction to date^15–17^. Therefore. further research to advance the development of the π-junction toward practical application are necessary.

Niobium (Nb) is often used as a superconducting material for π-junctions. From the viewpoint of superconducting logic circuits application, niobium nitride (NbN) is an attractive material, as it provides a high operating frequency of up to 1.2 THz and an operating temperature of 10 K, which cannot be achieved with Nb electrodes^18^. In addition, its large superconducting gap of 5.2 meV can suppress noise caused by quasiparticle excitation in the junctions, which is necessary for enhancing the coherence time of qubits^19^. For NbN-based ferromagnetic Josephson junctions, the ferromagnetic CuNi interlayer has been studied, and a π-phase shift in NbN/CuNi/NbN junctions has been demonstrated^20,21^. However, the CuNi-alloy contains Ni magnetic clusters and shows strong spin-flip scattering; thus, the Josephson critical current of the junctions with the CuNi interlayer decays quickly with increasing CuNi thickness^14,20^. On the contrary, in PdNi interlayers, Josephson critical currents have been observed over a wider range of PdNi thicknesses in Nb-based junctions, even in those using a 100-nm-thick PdNi interlayer^22–24^. From the viewpoint of device applications, the PdNi interlayer also provides advantages for NbN-based ferromagnetic Josephson junctions, including easier control over the critical currents and ensuring the π-state of junctions in large-scale circuits, even if there are spatial and/or run-to-run variations in PdNi thickness.

In this work, we studied NbN-based Josephson junctions with a diluted ferromagnetic Pd_89_Ni_11_ interlayer (NbN/PdNi/NbN junctions) and demonstrated their π-phase shift for the first time. We observed a non-monotonic dependence of critical currents (*I*_c_) on the PdNi layer thickness, indicating phase transition between the 0- and π-states owing to oscillation of the superconducting order parameter in the PdNi ferromagnetic interlayers. From the fitting of the experimental results based on an expression derived from the Usadel equation, we found that the spin-flip scattering degree in the PdNi interlayer was much smaller than that in the CuNi interlayer, leading to a longer decay length of the Josephson critical current. Based on these results, superconducting quantum interference devices (SQUIDs) were fabricated with SIS and SFS junctions. A clear half-flux-quantum shift was observed in the dependence of the critical current on the external magnetic field, thus providing evidence of the π-state.

### Fabrication and experimental setup

The MgO (100) was used as a substrate for NbN (100) film growth^25,26^. First, an NbN/PdNi/NbN trilayer was deposited in a multi-chamber sputtering system. The system was equipped with two separate sputtering chambers for NbN and PdNi films, in which the chambers were connected through a load lock chamber. A 200-nm-thick NbN was deposited as a base layer via reactive direct current (dc)-sputtering of an Nb target under an argon (Ar) and nitrogen gas atmosphere^20^. Then, the sample was transferred to the other chamber without breaking the vacuum, for deposition of the PdNi barrier by dc-sputtering with a Pd_89_Ni_11_ target. The thickness of the PdNi layer ranged from 10 to 40 nm by controlling the sputtering time. Next, the sample was sent back to the sputtering chamber to deposit the 200-nm-thick counter electrode NbN layer. All the sputtering processes are conducted at room temperature without heating, so the crystal or magnetic structure of PdNi film will not be affected by the high-temperature deposition.

**Figure 1:**
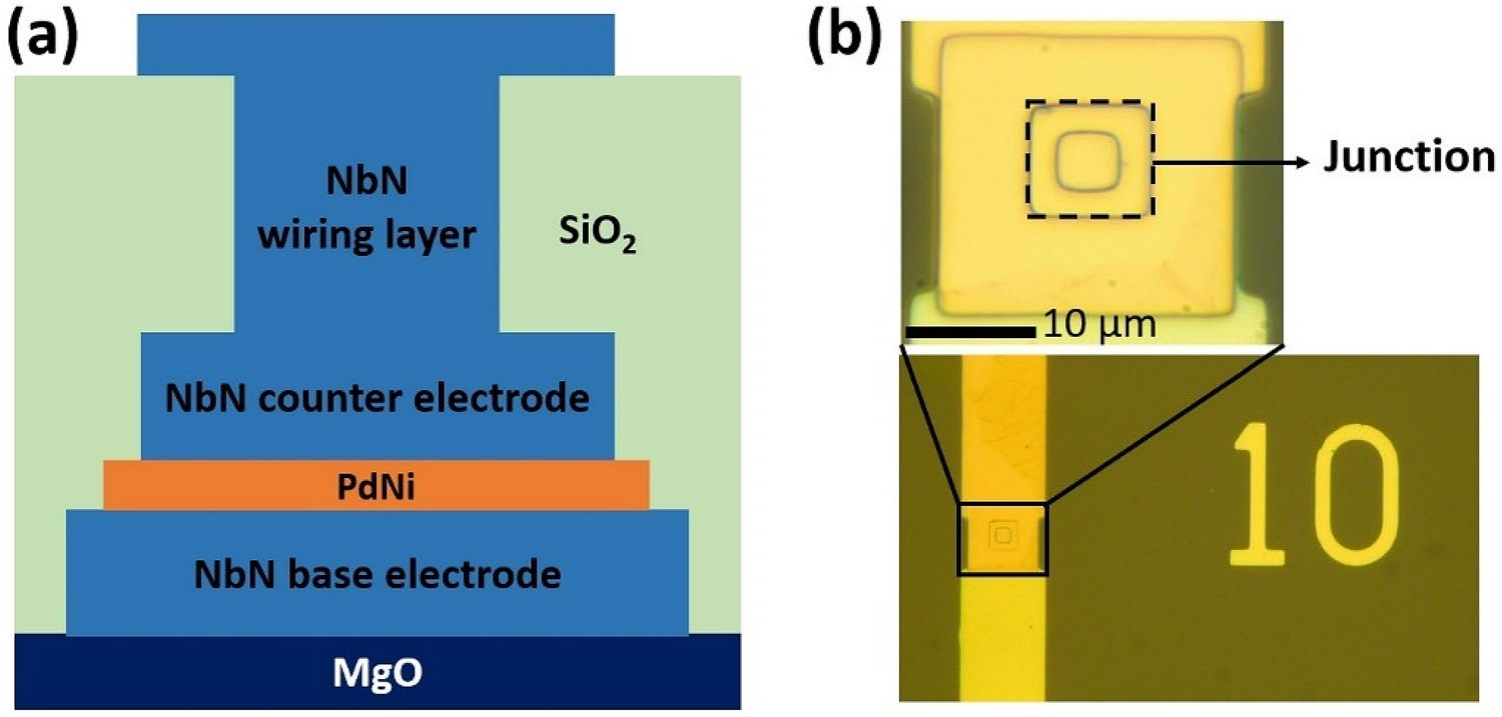
(**a**) Schematic cross-sectional view of the NbN/PdNi/NbN junction on an MgO substrate. (**b**) Microscope image of 10 × 10 µm^2^ junction.

The junctions were patterned in a square shape (size: 10 × 10 µm^2^) using conventional photolithography, and the counter electrode NbN layer was etched via reactive ion etching (RIE) with CF_4_ gas. The PdNi layer was fabricated by physical etching with Ar gas. Then, the base NbN electrode was patterned and etched by RIE. A 300-nm-thick SiO_2_ layer was deposited for isolating the base and NbN counter electrodes. The contact hole was created in the SiO_2_ layer and etched using RIE with CHF_3_ gas. Finally, a 450-nm-thick wiring NbN layer was deposited and patterned. Figure 1a,b show a schematic cross-sectional view and microscope image of the junction, respectively.

In addition to the single NbN/PdNi/NbN SFS junctions, we fabricated SQUID structures including two SIS junctions with an SFS junction, to determine the fabricated junction was in the π-state. First, the NbN-based SIS (NbN/AlN/NbN) junctions were fabricated. Then, the SFS junction was constructed to form the SQUID structure^21^. Although the fabrication process of the SFS junction was nearly the same as that for the single junction, the junction part was defined by the lift-off technique to avoid damage to the underlying structure, including the SIS junctions.

The current–voltage (*I*–*V*) characteristics of the junctions and magnetic field dependence of the critical currents of the SQUIDs were measured at 4.2 K using a magnetically shielded cryostat filled with liquid helium.

## Results and discussion

**Figure 2:**
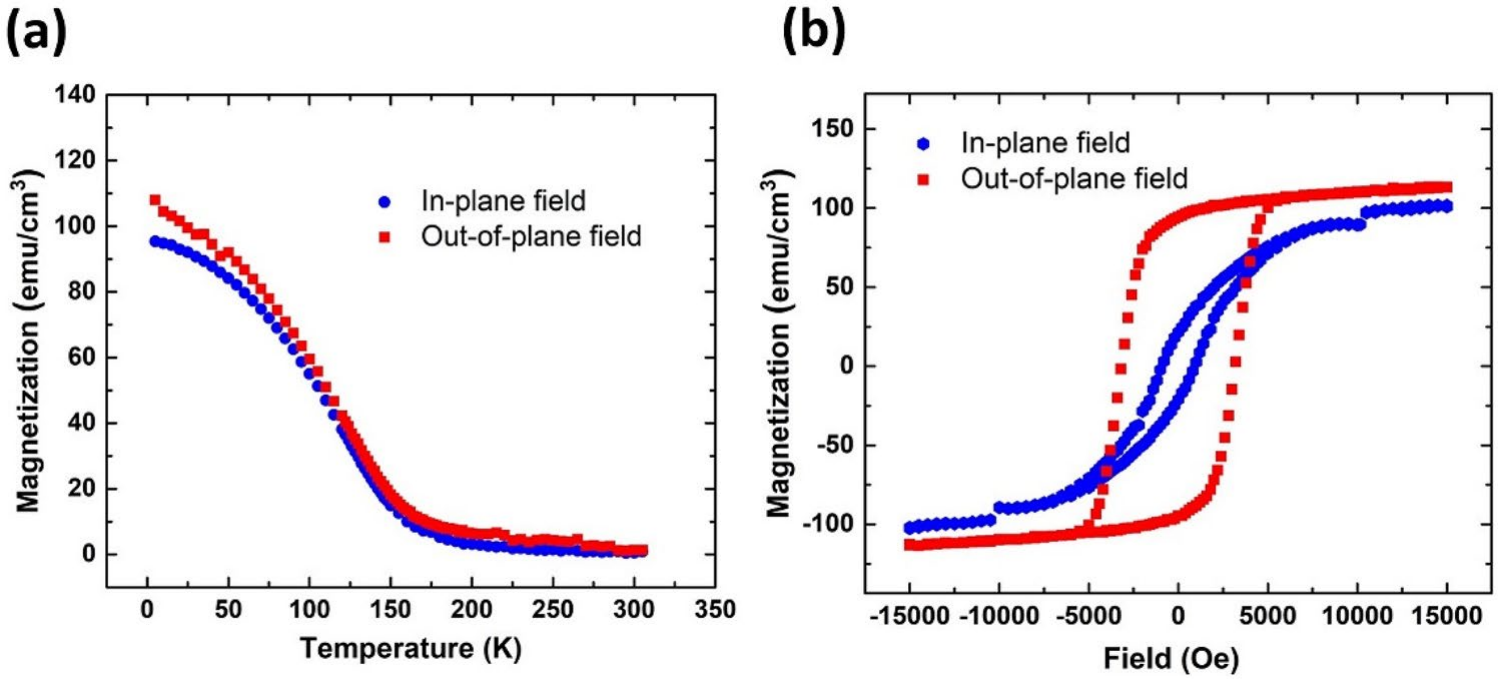
(**a**) Temperature-dependent magnetization (*M*–*T*) of a 35-nm-thick PdNi film at 10,000 Oe. (**b**) Magnetic field-dependent magnetization of the 35-nm-thick PdNi film at 4.2 K.

To investigate the magnetic properties of the PdNi interlayer, we deposited a 35-nm-thick PdNi film on a Si substrate and measured the temperature and magnetic field dependences of the magnetization using a commercial magnetic properties measurement system (MPMS; Quantum Design). Figure 2a shows the temperature dependence of the magnetization (*M–T*) for in-plane and out-of-plane magnetic fields at 10,000 Oe. To eliminate the signal of the substrate, we measured an Si substrate having the same size as that used for PdNi growth and subtracted the measurements from the data of the PdNi/Si sample. We confirmed that the jump of the magnetization around 10,000 Oe for the in-plane field was originated from the Si substrate. The Curie temperature (*T*_Curie_) was approximately 163 K both for in-plane and out-of-plane magnetic fields (Fig. 2a). Figure 2b shows the magnetic field dependences of the magnetization (*M–H*). From the *M*–*H* curves, the saturation magnetization (*M*_sat_) of the PdNi film for the out-of-plane field was determined as 120 emu/cm^3^. The obtained values of *T*_Curie_ and *M*_sat_ indicated that the Ni concentration of the deposited PdNi film was around 11%, which was the same as that of the PdNi target^24,27^. In a previous study of NbN/CuNi/NbN junctions, the Ni concentration obtained from *T*_Curie, CuNi_ = 200 K was ~ 59%, which was higher than that of the CuNi target (53%)^20^. The *M*–*H* curve showed clear out-of-plane magnetic anisotropy. For ferromagnetic thin films, the shape anisotropy usually dominates the magnetocrystalline anisotropy, resulting in in-plane anisotropy. However, in very thin films, the magnetic surface energy may be dominant, leading to out-of-plane anisotropy^28^. To date, the out-of-plane anisotropy of PdNi thin films with a thickness of up to 60 nm has been reported, as well as that for CuNi films with a thickness of 34 nm^23,29^. It is expected that the stray field from the PdNi interlayer could be reduced in SFS junctions compared to those related to in-plane anisotropy films^30^.

**Figure 3:**
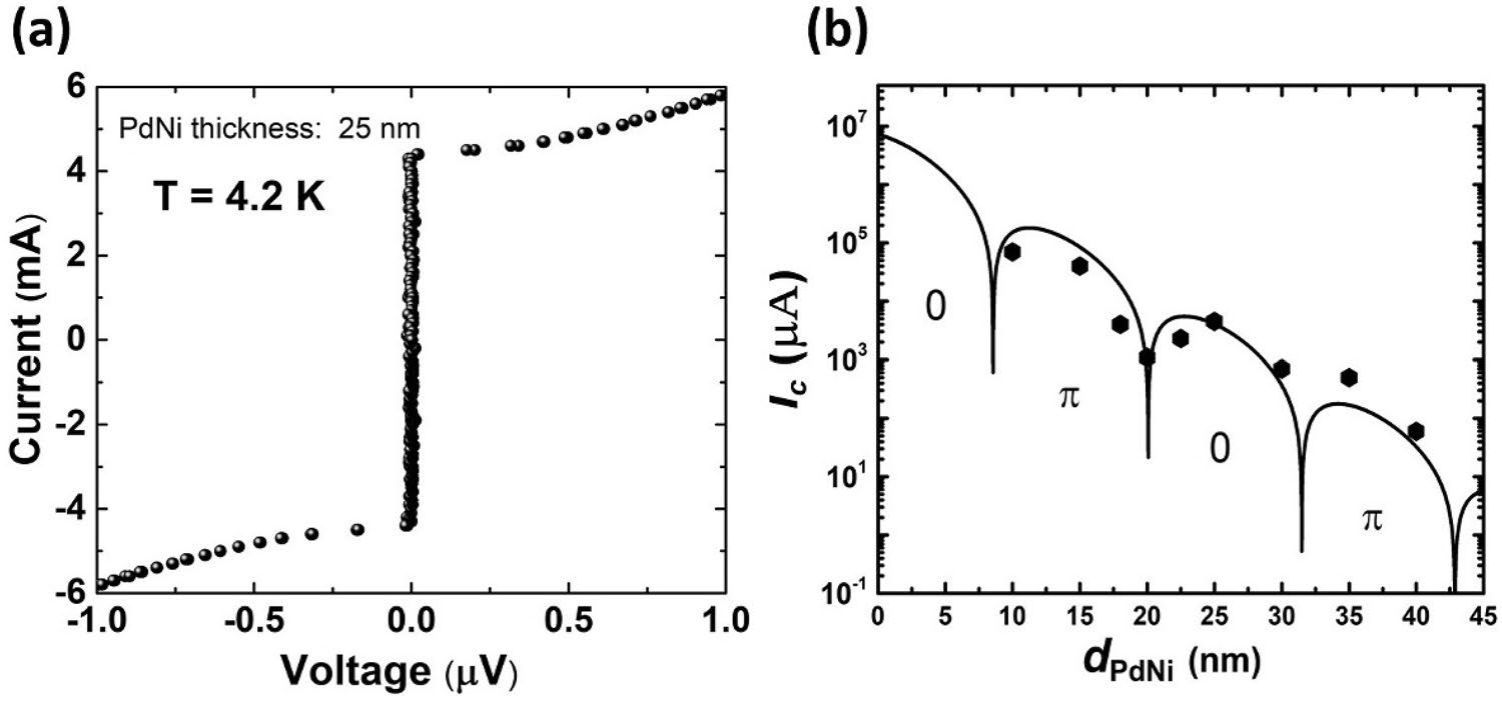
(**a**) Current–voltage characteristics of a 10 × 10 µm^2^ junction with a 25-nm-thick PdNi interlayer measured at 4.2 K. (**b**) Dependence of critical currents on the thickness of the PdNi interlayer. The solid line denotes the theoretical fitting of the data, where 0 and π indicate the expected 0 and π regions based on the fitting curve, respectively.

Next, we measured the *I*–*V* characteristics of the NbN/PdNi/NbN junctions at 4.2 K. Figure 3a shows the *I*–*V* characteristics of the junction with a 25-nm-thick PdNi interlayer. Overdamped *I*–*V* characteristics, which are typical for SFS junctions, were observed^20^. The normal resistance of the junction (*R*_*n*_) is 256 µΩ with 25-nm-thick PdNi. This value is higher than that extracted from the resistivity of a single Pd_89_Ni_11_ film (~ 90 µΩ)^31^, which may be due to the interface resistance arising in the non-epitaxial NbN counter-layer grown on PdNi. However, this interface resistance is much smaller than that reported for NbN/CuNi/NbN junctions^32^, in which the interface resistance was seven-fold larger than the resistance extracted from the resistivity of a single CuNi film.

Figure 3b shows the dependence of Josephson critical current (*I*_c_) on the PdNi thickness. The obtained *I*_c_ (symbols) showed non-monotonic dependence, which may indicate a phase transition between 0- and π-states. To analyze the experimental data, we considered the following theoretical expression of the critical currents in diffusive SFS junctions, derived from the Usadel equation^14,20^.

1 $$ I_{c} \left( {d_{F} } \right) = I_{c0} \left( {\frac{T}{{T_{c} }}} \right){\text{Re}} \left( {\mathop \sum \limits_{n = 0}^{\infty } \frac{{ {\mathcal{F}}\left( n \right)q_{1} \left( n \right)\exp \left[ { - \frac{{q_{1} \left( n \right) d_{F} }}{{\xi_{F} }}} \right]}}{{\left[ { \sqrt {q_{2} \left( n \right){\mathcal{F}}\left( n \right) + 1} + 1} \right]^{2} }}} \right), $$

where *I*_*c0*_ is a constant prefactor, *T*_*c*_ = 15 K is the critical temperature of NbN, *T* = 4.2 K is the measurement temperature, and *ξ*_*F*_ is the coherence length in the ferromagnetic interlayer. *n* is an integer, ${q}_{1}(n)$ = $\sqrt {2\left( {i + \alpha + \tilde{\omega }_{n} } \right)}$, ${q}_{2}(n)$ = (*i* + $\tilde{\omega }_{n}$)/(*i* + $\alpha$  +  $\tilde{\omega }_{n}$), and $\mathcal{F}\left(n\right)$ = ${\Delta }^{2} (T)$/[${\upomega }_{n}$ + $\sqrt{{\omega }_{n}^{2}+ {\Delta (T)}^{2}}$]^2^. Δ(*T*) is the superconducting gap, $\tilde{\omega }_{n}$ = ${\upomega }_{n}$/*E*_*ex*_ = π(2*n* + 1)*k*_*B*_*T*/*E*_ex_, and *α* = ℏ/(*τ*_s_*E*_*ex*_), where *E*_*ex*_ is the exchange energy and *τ*_s_ is the spin-flip scattering time in the ferromagnetic layer. *k*_*B*_ is Boltzmann’s constant and ℏ is the reduced Plank’s constant. The parameter *α* indicates the degree of spin-flip scattering. In Eq. (1), the free fitting parameters are *E*_*ex*_, *ξ*_*F*_, *α*, and *I*_*c0*_. The solid line in Fig. 3b indicates the curve fitted to the experimental data. From the fitting, we obtained the values as *E*_*ex*_/*k*_*B*_ = 175 K (*E*_*ex*_ $\approx$ 15 meV), *ξ*_*F*_ = 3.5 nm, *α* = 0.1, and *I*_*c0*_ = 1.8 × 10^8^ µA. The values of *E*_*ex*_ $\approx$ 15 meV and Δ = 5 meV indicate our junctions fulfill the diffusive limit of Eq. (1), since *E*_*ex*_, Δ ˂˂ ℏ/*τ*_e_ = 78 meV, where *τ*_e_ ≈ 8.4 × 10^–15^ s is the electron collisions time estimated from the resistivity and electron mean-free path (*l*_e_) of PdNi^23^.

In order to evaluate the relevance of Eq. (1) when apply to our data, we estimated the interface transparency *Ƭ* of PdNi junctions using the relation^33^: *ϒ*_B_ = $\frac{2}{3}$ $\frac{{l}_{e}}{{\xi }^{*}}$ $\frac{{1 - {\text{T}}}}{{\text{T}}}$. Here, the parameter *ϒ*_B_ = (*R*_B_*S*/*ρ*_F_ξ*), with *R*_B_ is interface resistance per unit area, *S* is the SFS junction area, *ρ*_F_ is the resistivity of PdNi layer and ξ* = $\sqrt{\hslash D/{2\pi k}_{B}{T}_{c}}$. In our junction, *R*_B_ = 83 µΩ for 10 × 10 µm^2^ junction (estimated from junction’s resistance and resistance extracted from resistivity of PdNi film), *ρ*_F_ = 36 µΩ cm, ξ* = 4.8 nm (calculated from ξ*/*ξ*_*F*_ ratio), so *ϒ*_B_ = *R*_B_*S*/*ρ*_F_ξ* ≈ 4.8. The electron mean-free path *l*_e_ ≈ 4.7 nm was estimated using the relation *ρ*_F_*l*_e_ = 1.7 fΩ m^2^, which has been reported for PdNi^23^. Finally, we obtained the interface transparency *Ƭ* = 0.12. The condition *Ƭ* = 0 corresponds to a completely reflecting interface, whereas *Ƭ* = 1 corresponds to a perfect transparent boundary. Experimentally, it is not practical to get the perfect transparency with *Ƭ* = 1. Our result of *Ƭ* = 0.12, however, equal to the transparency value calculated for CuNi junctions (with *ϒ*_B_ = 0.52, *l*_e_ = 1 nm, ξ* = 9.4 nm) in the reference^34^, where Eq. (1) has been used with good approximation. Therefore, our PdNi junctions fulfill the condition of interface transparency for this expression.

Here we discuss the obtained fitting parameters. First, the exchange energy *E*_*ex*_ of 15 meV is in good agreement with that for the Pd_89_Ni_11_ alloy in a previous report^27^. The value of *ξ*_*F*_ = 3.5 nm is also consistent with values from the literature: *ξ*_*F*_ = 3.3–4.0 nm for Nb-based SFS junctions with a PdNi alloy with similar Ni concentrations^23,27,35^. Next, we compared the obtained parameters with those for the NbN/Cu_40_Ni_60_/NbN junction^20^. The value of *ξ*_*F*_ (3.5 nm) was larger than that of 1.9 nm reported for NbN/CuNi/NbN junctions, due to the smaller *E*_*ex*_ and larger diffusion constant *D* in ${\xi }_{F}=\sqrt{\hslash D/{E}_{ex}}$ for the NbN/PdNi/NbN junctions^13^. Regarding the degree of spin-flip scattering, we found that the value of *α* was 0.1, which is much smaller than that of 0.8 for the NbN/CuNi/NbN junction^20^. We calculated the decay length (*ξ*_*F1*_) and oscillation length (*ξ*_*F2*_) of the Josephson critical current *I*_c_(*d*_*F*_) using the equation *ξ*_*F1(F2)*_ (*T*) = *ξ*_*F*_/$\sqrt {\sqrt {1 + (\tilde{\omega }_{n} + \alpha )^{2} } \pm \left( {\tilde{\omega }_{n} + \alpha } \right)}$, and obtained the following^20^: *ξ*_*F1*_(0) = 3.3 nm and *ξ*_*F2*_(0) = 3.9 nm. Owing to the smaller *α* and larger *ξ*_*F*_, the decay length *ξ*_*F1*_ in the NbN/PdNi/NbN junction became much larger than that of 1.3 nm in the CuNi junctions. Furthermore, *ξ*_*F1*_(0) and *ξ*_*F2*_(0) were comparable due to the small value of *α*, and the oscillation length *ξ*_*F2*_(0) was also larger than that of the 2.8 nm reported for NbN/CuNi/NbN junctions^20^. This small value of *α*, i.e., weak spin-flip scattering, in the PdNi interlayer may originate from better magnetic uniformity with fewer magnetic impurities (e.g., clusters). It is known that, in CuNi films, ferromagnetism originates from clusters of at least eight Ni atoms, due to statistical concentration fluctuations^36^. On the other hand, in the PdNi film, the ferromagnetism is caused not only by the diluted Ni atoms but also by paramagnetic Pd atoms. This leads to homogeneous ferromagnetism in PdNi rather than a matrix with ferromagnetic clusters in CuNi. From a device application perspective, the longer *ξ*_*F1*_ and *ξ*_*F2*_ obtained in the PdNi junction are attractive, as it becomes easier to fabricate a π-junction with the desired critical current, even under the conditions of process variability and/or spatial variation of the PdNi thickness. Lastly, the obtained value of 1.8 × 10^8^ µA for *I*_*c0*_ is also comparable to that calculated from the actual physical parameters of the fabricated junctions (*T*_*c*_ and *R*_*n*_) using the expression obtained from the Usadel equation^14,20^.

It is necessary to discuss the role of the spin–orbit scattering in PdNi interlayer since Pd is a heavy metal. In SFS junctions, the spin–orbit and spin-flip scattering have a similar effect on *ξ*_*F1*_ and *ξ*_*F2*_. If the spin–orbit scattering rate is larger than *E*_ex_/ℏ, the oscillations of the junction’s critical currents disappear completely^23^. Our results in Fig. 3b, however, shows a clear oscillation of critical currents, so the spin–orbit scattering rate was expected to smaller than the limit above.

From the fitting of the experimental data, we can expect a π-state region with a PdNi thickness in the range of 8–20 nm, as shown in Fig. 3b. To determine whether the π-state actually emerged, we selected SFS junctions with 10- and 15-nm-thick PdNi interlayers, and fabricated and characterized SQUID structures with these junctions as follows.

**Figure 4:**
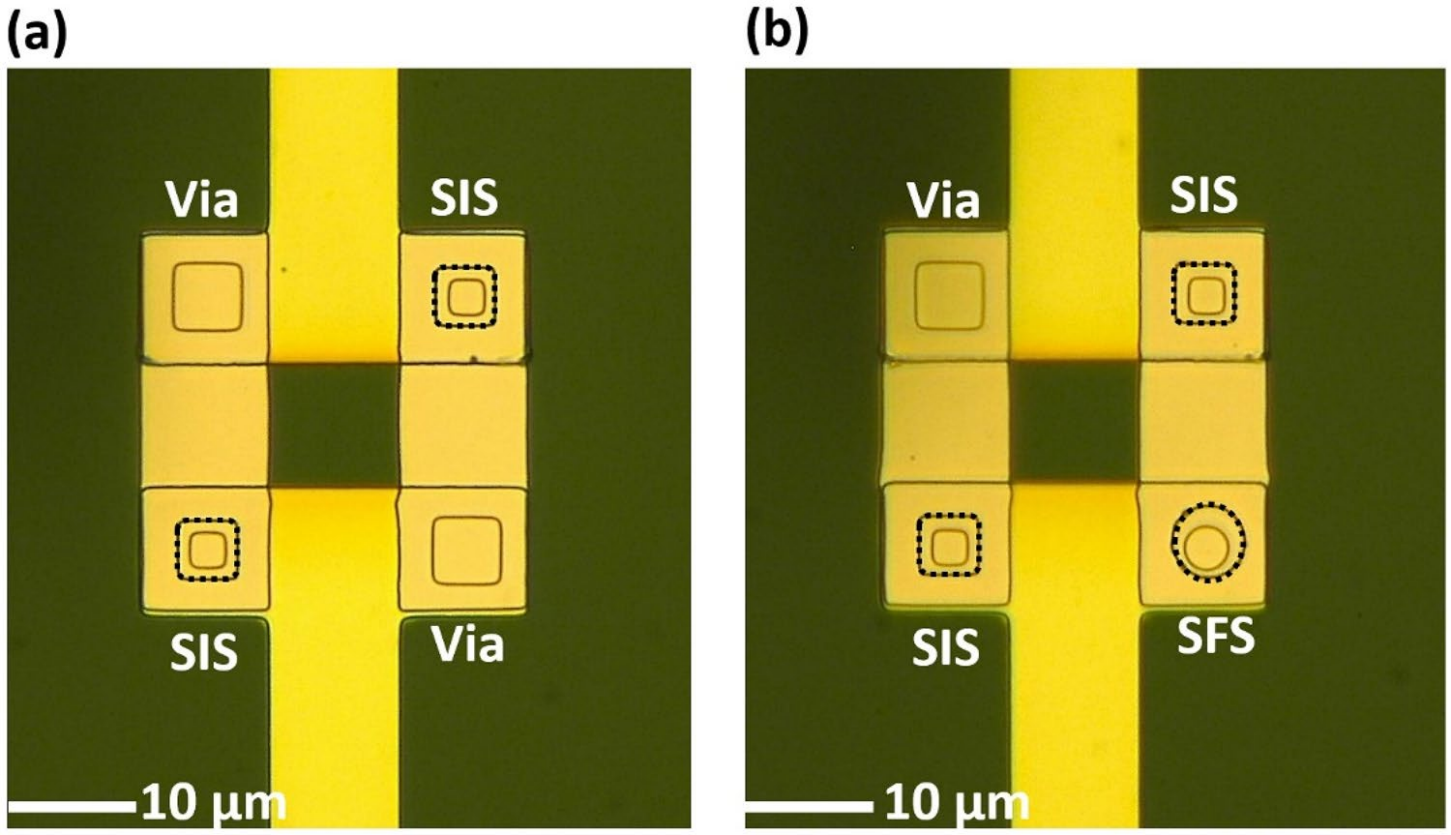
Microscope images of (**a**) a direct-current superconducting quantum interference device (dc-SQUID) and (**b**) a superconductor/ferromagnetic/superconductor (SFS)-SQUID with superconductor/insulator/superconductor (SIS) and SFS junctions, as denoted by the dotted squares and circles, respectively.

On a chip, we fabricated two SQUIDs: a conventional SQUID with two SIS junctions (dc-SQUID) and a SQUID with two SIS junctions and one SFS junction (an SFS-SQUID), as shown in Fig. 4a,b, respectively. The SIS junctions were NbN/AlN/NbN junctions with critical currents of 15 µA and a size of 5 × 5 µm^2^, giving a critical current density of 60 A/cm^2^, which is typically used for superconducting qubits^19^. The SFS junctions were circular NbN/PdNi/NbN junctions with a diameter of 5 µm. The critical currents of the SFS junctions were 3.0 and 2.9 mA for 10- and 15-nm-thick PdNi, respectively. The critical current density of the SFS junctions with both 10- and 15-nm-thick PdNi layers was around 15 kA/cm^2^, which was about three-fold higher than that of the SIS junction. Therefore, the SFS junction worked as a passive *π*-phase shifter, even with its much smaller size^10,21^. For the dc-SQUID, when the inductance of the loop is negligible, the magnetic field-dependent critical currents can be described by the equation: ${I}_{c,dc-SQUID}$ = 2 ${I}_{c,SIS}$ │cos(π$\frac{{\Phi_{{{\text{ext}}}} }}{{\Phi_{0} }}$)│. Here, *Ф*_*ext*_ is the externally applied magnetic field and *Ф*_0_ is the single flux quantum. In the case of the SFS-SQUID, when the *π*-junction is integrated into the loop having a critical current higher than that of the SIS junctions (${I}_{c,\pi }$ > ${I}_{c,SIS}$), it plays the role of a *π*-phase shifter, resulting in the *π*-shifted relation^37,38^: ${I}_{c,SFS-SQUID}$ = 2 ${I}_{c,SIS}$│sin(π $\frac{{\Phi_{{{\text{ext}}}} }}{{\Phi_{0} }}$)│.

**Figure 5:**
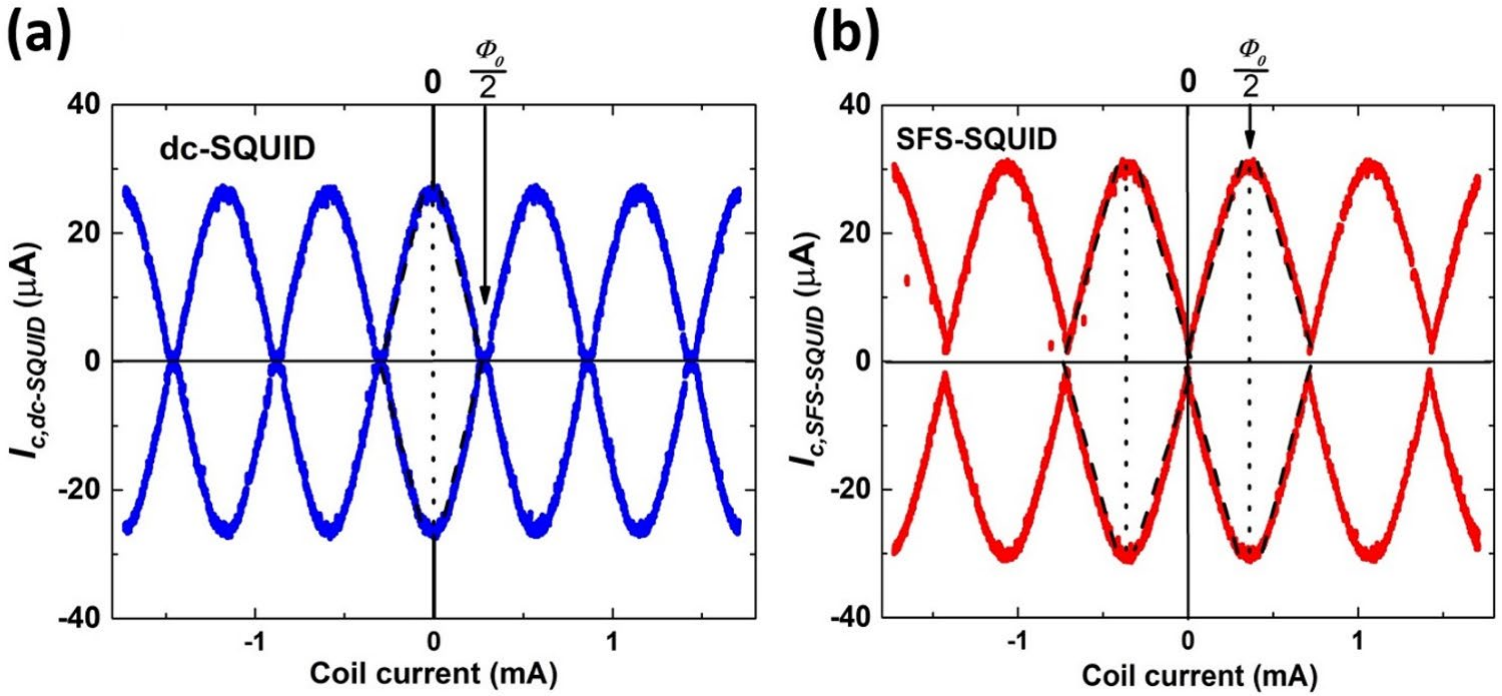
Magnetic field-dependent critical currents of (**a**) the dc-SQUID and (**b**) SFS-SQUID. The arrows indicate the field value at a half-flux-quantum.

Figure 5a,b show the dependences of the positive and negative critical currents on the magnetic coil currents for the dc- and SFS-SQUIDs with a 10-nm-thick PdNi interlayer, respectively. The dashed lines represent the border for the vortex state and intersection between dotted lines (two connected peaks of ${I}_{c,dc-SQUID}$), and the horizontal axis indicates the quantum number of the SQUID. Our results show that ${I}_{c,dc-SQUID}$ = 2 ${I}_{c,SIS}$ at zero magnetic coil current for the dc-SQUID, as expected. When the coil current increased (or decreased), local minima appeared in ${I}_{c,dc-SQUID}$, corresponding to the half-flux quantum value (± *Ф*_*0*_/2) or a *π*-phase difference between the two arms of the SQUID loop. The peaks and dips in the modulation pattern can be understood as constructive and destructive interference of the currents in the two arms of the SQUID, respectively. At the half-flux quantum value of the magnetic fields, the ${I}_{c,dc-SQUID}$ drops to around zero due to the small inductance of the SQUID loop. We also calculated the inductance of the SQUID loop from the geometric inductance (*L*_*g*_ = 1.25 *µ*_*0*_*d* ≈ 15.7 pH) and kinetic inductance (*L*_*k*_ = *µ*_0_λ^2^*l*/*wh* ≈ 0.3 pH). Here, *µ*_0_ is the vacuum permeability, *d* is arm’s length of SQUID, λ is the penetration depth of NbN, *l* is total loop’s length, *w* is arm’s width, and *h* is thickness of superconducting film^37^. The estimated value of the loop inductance was *L* = 16.0 pH, giving a screening parameter ${\beta }_{L}$ = $\frac{{2LI_{c,SIS} }}{{\Phi_{0} }}$  = 0.2. There is a relationship between *β*_*L*_ and the depth of the modulation pattern, at *β*_*L*_ = 0.2, only 10% of the maximum ${I}_{c,dc-SQUID}$ occurs, corresponding to around 3 µA for this dc-SQUID^37^. This very small critical current is usually suppressed by external noise, thus, giving the results shown in Fig. 5a.

On the other hand, for the SFS-SQUID shown in Fig. 5b, ${I}_{c,SFS-SQUID}$ reached its minimum at zero magnetic coil current. As the coil current increased (or decreased), the local maxima of ${I}_{c,SFS-SQUID}$ = 2 ${I}_{c,SIS}$ appeared at *Ф*_*ext*_ =  ± *Ф*_0_/2. The intersections between the horizontal axis and dotted lines were equally shifted from the origin by a half period of the modulation pattern. Compared to the dc-SQUID, a clear half-flux-quantum shift was observed in the modulation pattern of the SFS-SQUID^21,38^. A half-flux-quantum shift was also observed in the SFS-SQUID with a 15-nm-thick PdNi interlayer. These results provide evidence of the *π*-state in SFS junctions. In Fig. 5b, a reduction of the modulation depth in the SFS-SQUID compared to the dc-SQUID can be seen, due to the increased inductance of the SQUID loop caused by the kinetic inductance of the SFS junction.

We also fabricated the SFS-SQUIDs with Nb/AlO_*x*_/Al/Nb junctions. In these samples, the Nb/AlO_*x*_/Al/Nb junctions were fabricated on Nb base electrode by using the standard fabrication process of clean room for analog and digital superconductivity (CRAVITY) in the National Institute of Advanced Industrial Science and Technology (AIST) Japan. Then, we fabricated the NbN/PdNi/NbN junctions with 15- and 35-nm-thick PdNi on the polycrystalline Nb base layer of the SQUIDs. A *π*-phase shift was observed in the modulation pattern of these SFS-SQUIDs. The *π*-phase shift demonstrated by the NbN-based SFS-SQUIDs and Nb four-layer structure SFS-SQUIDs expands its application fields.

## Conclusions

In conclusion, we studied NbN/Pd_89_Ni_11_/NbN junctions with a PdNi thickness of 10–40 nm and observed non-monotonic behavior in the thickness-dependent critical currents of the junctions, indicating the oscillation of the superconducting order parameter due to the exchange interaction in the PdNi ferromagnetic layer. Spin-flip scattering in the PdNi junctions was significantly weaker than that in CuNi junctions, leading to a longer decay length. This provides advantages for the fabrication and performance of the device, such as ease in controlling the critical currents and ensuring working π-junctions for large-scale circuits, even with spatial variation in the PdNi thickness. Lastly, we fabricated and measured the magnetic field-dependent critical currents of dc-SQUIDs and SFS-SQUIDs for demonstrating the π-state of junctions. A half-flux quantum shift was observed in the modulation pattern of SFS-SQUIDs compared to the dc-SQUID, indicating the presence of π junctions. These results not only provide an important understanding of the physics of ferromagnetic Josephson junctions but also an initial step toward practical application.

## Data Availability

All data included in this study are available upon request by contact with the corresponding author.

## References

[1] Josephson BD (1962). Possible new effects in superconductive tunnelling. Phys. Lett..

[2] Bulaevskiǐ LN, Kuziǐ VV, Sobyanin AA (1977). Superconducting system with weak coupling to the current in the ground state. J. Exp. Theor. Phys. Lett..

[3] Kjaergaard M, Schwartz ME, Braumuller J, Krantz P, Wang JI-J, Gustavsson S, Oliver WD (2020). Superconducting Qubits: Current State of Play. Annu. Rev. Condens. Matter Phys..

[4] Yan F (2016). The flux qubit revisited to enhance coherence and reproducibility. Nat. Comm..

[5] Yoshihara F, Harrabi K, Niskanen AO, Nakamura Y, Tsai JS (2006). Decoherence of Flux Qubits due to 1/f Flux Noise. Phys. Rev. Lett..

[6] Yamashita T, Tanikawa K, Takahashi S, Maekawa S (2005). Superconducting π Qubit with a ferromagnetic Josephson junction. Phys. Rev. Lett..

[7] Yamashita T, Takahashi S, Maekawa S (2006). Superconducting π qubit with three Josephson junctions. Appl. Phys. Lett..

[8] Kato T, Golubov AA, Nakamura Y (2007). Decoherence in a superconducting flux qubit with a π-junction. Phys. Rev. B.

[9] Ustinov AV, Kaplunenko VK (2003). Rapid single-flux quantum logic using π-shifters. J. Appl. Phys..

[10] Li F, Takeshita Y, Hasegawa D, Tanaka M, Yamashita T, Fujimaki A (2021). Low-power high-speed half-flux-quantum circuits driven by low bias voltages. Supercond. Sci. Technol..

[11] Kamiya, T., Tanaka, M., Sano, K. & Fujimaki, A. Energy/Space-Efficient Rapid Single-Flux-Quantum Circuits by Using π-Shifted Josephson Junctions. *IEICE Trans. Elec.* **E101.C**, 385–390 (2018).

[12] Ryazanov VV, Oboznov VA, Rusanov AY, Veretennikov AV, Golubov AA, Aarts J (2001). Coupling of two superconductors through a ferromagnet: Evidence for a π junction. Phys. Rev. Lett..

[13] Kontos T, Aprili M, Lesueur J, Genet F, Stephanidis B, Boursier R (2002). Josephson junction through a thin ferromagnetic layer: Negative Coupling. Phys. Rev. Lett..

[14] Buzdin AI (2005). Proximity effects in superconductor-ferromagnet heterostructures. Rev. Moder. Phys..

[15] Feofanov AK (2010). Implementation of superconductor/ferromagnet/superconductor π-shifters in superconducting digital and quantum circuits. Nat. Phys..

[16] Hasegawa D, Takeshita Y, Li F, Sano K, Tanaka M, Yamashita T, Fujimaki A (2021). Demonstration of interface circuits between half- and single- flux- quantum circuits. IEEE Trans. Appl. Supercond..

[17] Takeshita Y, Li F, Hasegawa D, Sano K, Tanaka M, Yamashita T, Fujimaki A (2021). High-speed memory driven by SFQ pulses based on 0-π SQUID. IEEE Trans. Appl. Supercond..

[18] Makise K, Terai H, Miki S, Yamashita T, Wang Z (2013). Design and fabrication of all-NbN SFQ circuits for SSPD signal processing. IEEE Trans. Appl. Supercond..

[19] Kim S, Terai H, Yamashita T, Qiu W, Fuse T, Yoshihara F, Ashhab S, Inomata K, Semba K (2021). Enhanced-coherence all-nitride superconducting qubit epitaxially grown on Si Substrate. Commun. Mater..

[20] Yamashita T, Kawakami A, Terai H (2017). NbN-based ferromagnetic 0 and π Josephson junctions. Phys. Rev. Appl..

[21] Yamashita T, Kim S, Kato H, Qiu W, Semba K, Fujimaki A, Terai H (2020). π phase shifter based on NbN-based ferromagnetic Josephson junction on a silicon substrate. Sci. Rep..

[22] Iannone G, Zola D, Armenio AA, Polichetti M, Attanasio C (2007). Electrical resistivity and magnetic behavior of PdNi and CuNi thin films. Phys. Rev. B.

[23] Khaire TS, Pratt WP, Birge NO (2009). Critical current behavior in Josephson junctions with the weak ferromagnet PdNi. Phys. Rev. B.

[24] Wild, G. H. Josephson Junctions with Ferromagnetic Interlayer (Ph.D. Thesis, Technische Universität München, 2012).

[25] Wang Z, Terai H, Qiu W, Makise K, Uzawa Y, Kimoto K, Nakamura Y (2013). High-quality epitaxial NbN/AlN/NbN tunnel junctions with a wide range of current density. Appl. Phys. Lett..

[26] Nakamura Y, Terai H, Inomata K, Yamamoto T, Qiu W, Wang Z (2011). Superconducting qubits consisting of epitaxially grown NbN/AlN/NbN Josephson junctions. Appl. Phys. Lett..

[27] Cirillo C, Ilyina EA, Attanasio C (2011). Static and dynamic properties of the vortex lattice in superconductor/weak ferromagnet bilayers. Supercond. Sci. Technol..

[28] Vaz CAF, Bland JAC, Lauhoff G (2008). Magnetism in ultrathin film structures. Rep. Prog. Phys..

[29] Ruotolo A, Bell C, Leung CW, Blamire MG (2004). Perpendicular magnetic anisotropy and structural properties of NiCu/Cu multilayers. J. Appl. Phys..

[30] Morini M, Slastikov V (2018). Reduced models for ferromagnetic thin films with periodic surface roughness. J. Nonlinear Sci..

[31] Ito H, Taniguchi S, Ishikawa K, Akaike H, Fujimaki A (2017). Fabrication of superconductor-ferromagnet-insulator-superconductor Josephson junctions with critical current uniformity applicable to integrated circuits. Appl. Phys. Express.

[32] Li F, Zhang H, Zhang L, Peng W, Wang Z (2018). Ferromagnetic Josephson junctions based on epitaxial NbN/Ni_60_Cu_40_/NbN trilayer. AIP Advance.

[33] Fominov, Ya. V., Chtchelkatchev, N. M. & Golubov, A. A. Nonmonotonic critical temperature in superconductor/ferromagnet bilayers. *Phys. Rev. B* **66**, 014507 (2002).

[34] Oboznov, V. A., Bol’ginov, V. V., Feofanov, A. K., Ryazanov, V. V. & Buzdin, A. I. Thickness Dependence of the Josephson Ground States of Superconductor-Ferromagnet-Superconductor Junctions. *Phys. Rev. Lett.* **96**, 197003 (2006). 10.1103/PhysRevLett.96.197003 16803133

[35] Cirillo C, Prischepa SL, Salvato M, Attanasio C, Hesselberth M, Aarts J (2005). Superconducting proximity effect and interface transparency in Nb/PdNi bilayers. Phys. Rev. B.

[36] Ododo JC, Coles BR (1977). The critical concentration for the onset of ferromagnetism in CuNi alloys. J. Phys. F: Metal Phys..

[37] Clarke, A. I. B. J. *The SQUID Handbook* (Fundamentals and Technology of SQUIDs and SQUID System, Germany) **I**, 46 (2004).

[38] Frolov, S. M. Current-phase relations of Josephson junctions with ferromagnetic barriers (Ph.D. Thesis, University of Illinois at Urbana-Champaign, 2005).

